# The estrogen receptor 1 gene affects bone mineral density and osteoporosis treatment efficiency in Slovak postmenopausal women

**DOI:** 10.1186/s12881-018-0684-8

**Published:** 2018-09-21

**Authors:** Vladimira Mondockova, Maria Adamkovicova, Martina Lukacova, Birgit Grosskopf, Ramona Babosova, Drahomir Galbavy, Monika Martiniakova, Radoslav Omelka

**Affiliations:** 1 Department of Botany and Genetics, Constantine the Philosopher University in Nitra, Nitra, Slovak Republic; 2 Institute of Zoology and Anthropology, Georg-August University, Göttingen, Germany; 3 Department of Zoology and Anthropology, Constantine the Philosopher University in Nitra, Nitra, Slovak Republic; 4 Private Orthopedic Ambulance, Nitra, Slovak Republic

**Keywords:** Osteoporosis, *ESR1* gene, Polymorphisms, BMD, Fractures, HT, Estradiol, Raloxifene

## Abstract

**Background:**

The study investigated the associations of rs9340799:A > G (XbaI) and rs2234693:T > C (PvuII) polymorphisms in the estrogen receptor 1 gene (*ESR1*) with femoral neck (BMD-FN) and lumbar spine bone mineral density (BMD-LS), biochemical markers of bone turnover, calcium and phosphate levels, fracture prevalence, and a response to two types of anti-osteoporotic therapy in postmenopausal women from southern Slovakia.

**Methods:**

We analysed 343 postmenopausal Slovak women (62.40 ± 0.46 years). The influence of rs9340799 (AA vs. AG + GG) and rs2234693 (TT vs. TC + CC) genotypes on BMD and biochemical markers was evaluated by covariance analysis adjusted for age and BMI. Binary logistic regression was used to evaluate the genotype effect on fracture prevalence. Pharmacogenetic part of the study included women who received a regular therapy of HT (17ß estradiol with progesterone; 1 mg/day for both; *N* = 76) or SERMs/raloxifene (60 mg/day; *N* = 64) during 48 months. The genotype-based BMD change was assessed by variance analysis for repeated measurements.

**Results:**

Women with AA genotype of rs9340799 had higher BMD-FN (+ 0.12 ± 0.57 of T-score) and BMD-LS (+ 0.17 ± 0.08 of T-score) in comparison with AG + GG. The rs2234693 polymorphism did not affect any of the monitored parameters. No effect of any *ESR1* polymorphisms was found on fracture prevalence. Both types of anti-osteoporotic therapy had a positive effect on BMD improvement in FN and LS sites. Considering the effect of the *ESR1* gene within the HT, the subjects with rs9340799/AA genotype showed worse response than those with GG genotype (− 0.26 ± 0.10 of BMD-FN T-score; − 0.35 ± 0.10 of BMD-LS T-score) and also with AG genotype (− 0.22 ± 0.08 of BMD-LS T-score). The rs2234693/TT genotype responded poorer in BMD-LS in comparison with TC (− 0.22 ± 0.08 of T-score) and CC (− 0.35 ± 0.09 of T-score). The effect of the *ESR1* gene on raloxifene therapy was reported only in BMD-LS. Subjects with rs9340799/AA genotype had a − 0.30 ± 0.11 of T-score worse response compared to AG genotype. The rs2234693/TT genotype showed − 0.39 ± 0.11 and − 0.46 ± 0.15 lower T-scores in comparison with TC and CC genotypes, respectively.

**Conclusions:**

The rs9340799 polymorphism may contribute to decreased BMD in postmenopausal women from southern Slovakia; however, this is not related to higher fracture prevalence. Concurrently, both polymorphisms affected a response to analysed anti-osteoporotic therapies.

**Electronic supplementary material:**

The online version of this article (10.1186/s12881-018-0684-8) contains supplementary material, which is available to authorized users.

## Background

Osteoporosis is a common disease, characterized by reduced bone mass, defects in the microarchitecture of bone tissue, and an increased risk of fragility fractures [1]. The presence of fractures together with bone mineral density (BMD) measurements forms the basis of diagnostic techniques that guide targeted intervention strategies. The etiology of osteoporosis is multifactorial in which a polygenic background is modulated by the integrated effects of hormonal, environmental and nutritional factors. Although many environmental factors play an important role in BMD variation, genetic influences account for 60–85% of individual variance. So far, genetic studies have revealed candidate genes included in the regulation of BMD and in the osteoporosis progression [2–4]. Estrogen deficiency represents a major mechanism of the rapid bone loss in postmenopausal women. Interactions of estrogens with the receptors in target cells of bone and other tissues regulate growth and bone development, acquisition of peak bone mass, bone metabolism and inhibition of bone loss [5]. Therefore, the gene encoding estrogen receptor 1, one of two mediators of estrogen action, has been considered as an important candidate for the determination of osteoporosis risk [6, 7]. The principal role of the *ESR1* gene in skeletal maintenance has recently been confirmed using mice with targeted deletion of *ESR1* from specific bone cells and their precursors. Lack of the estrogen receptor in osteoblast progenitor and precursor cells affected the periosteum while the absence of the receptor in differentiated osteoblasts, osteocytes, and osteoclasts resulted in reduced cancellous bone mass [8]. Genetic screening of the *ESR1* gene locus has revealed several polymorphic sites. The most widely studied are rs2234693:T > C (PvuII), rs9340799:A > G (XbaI) polymorphisms in intron I, and the (TA)n repeat polymorphism within the promoter region of the gene [6]. Several studies showed a relationship between low number of TA repeats and increased fracture risk or BMD in different populations [9, 10]. Within the rs2234693 and rs9340799 polymorphisms, the results have not always been consistent in different population analyses. However, despite conflicting results, associations of rs2234693 and rs9340799 polymorphisms with BMD have been found in some studies [4, 11–15].

Considering the mechanisms of drug action within specific treatment procedures, such as hormone therapy (HT) or selective estrogen receptor modulators (SERMs) application, the genetic variability in the *ESR1* gene may also have important pharmacogenetic implications. The HT is a treatment commonly used to relieve symptoms and some undesirable consequences of menopause including osteoporosis. Exogenous estrogens also belong to the primary osteoporosis prevention in postmenopausal women, as these agents reduce the risk of vertebral and hip fractures [16]. SERMs are used for prevention and treatment of postmenopausal osteoporosis and breast cancer prevention in high-risk postmenopausal women with osteoporosis [17]. Raloxifene, a member of SERMs, simulates estrogen action on the skeletal system through agonistic binding to estrogen receptors without the negative effects on breast and endometrium [18]. As in the case of association studies, the results of pharmacogenetic ones have not always been consistent. Positive effects of TT genotype on fracture risk [19] and BMD [12, 20, 21] were found in different populations. In addition to HT and SERMs, other currently approved therapies for osteoporosis include bisphosphonates (BPs), applications of vitamin D derivates, parathyroid hormone and teriparatide (a recombinant human parathyroid hormone), calcitonin, strontium ranelate, and anti-RANK ligand monoclonal antibodies [22]. Response to drugs can be affected by many factors, such as sex, age, ethnicity, lifestyle, and concomitant diseases or drug therapy. The individual variation of response to anti-osteoporotic treatments ranges from good to little response or nonresponse (estimated proportion from 5 to 10%), and it may be due to individual genetic factors or environmental influences that could interfere with drug dynamics and kinetics [23]. Common variations in the human genome are today considered as the most important cause of variable drug responses [18].

The aim of this study was to analyse the associations of rs2234693 and rs9340799 polymorphisms in the *ESR1* gene with BMD, biochemical markers of bone turnover, calcium and phosphate levels, fracture prevalence, and a response to two types of anti-osteoporotic therapy in postmenopausal women from southern Slovakia.

## Methods

### Studied population

Our study included 343 postmenopausal women from southern region of the Slovak Republic aged from 45 to 85 years (62.40 ± 0.46 years) and monitored under the basic diagnostic screening for osteoporosis. Women were selected according to strict inclusion criteria. We excluded women with serious internal, endocrine, chronic and hereditary diseases, patients treated with certain medicaments (glucocorticoids, hormones) and with previous antiosteoporotic treatment, obese women (BMI = 30.0 kg/m^2^ and above), women with a significant abuse (alcoholism, nicotinism, caffeinism), individuals with late-onset or premature menopause, and women with serious disturbances in the menstrual cycle. Clinical characteristics and parameters of the study population are shown in Table 1. The proportion of subjects with diagnosed osteoporosis accounted for 60.1% (*N* = 206) of all women.

**Table 1:** General characteristics of the studied groups of women

Variable	Total *N* = 343	HT study *N* = 76	Raloxifene study *N* = 64
Age (years)	62.40 ± 0.46	63.22 ± 1.00	65.30 ± 0.98
Body mass index (BMI)	27.60 ± 0.08	27.30 ± 0.18	27.64 ± 0.17
BMD-FN (T-score)	−1.79 ± 0.03	− 2.13 ± 0.04	−2.16 ± 0.06
BMD-FN (g/cm^2^)	0.65 ± 0.01	0.60 ± 0.01	0.60 ± 0.01
BMD-LS (T-score)	−2.37 ± 0.04	−2.87 ± 0.04	− 2.95 ± 0.05
BMD-LS (g/cm^2^)	0.73 ± 0.01	0.68 ± 0.01	0.67 ± 0.01
Bone isoenzyme of alkaline phosphatase (μkat/l)	0.56 ± 0.04	1.17 ± 0.11	0.68 ± 0.08
Osteocalcin (μg/l)	3.85 ± 0.05	3.92 ± 0.11	4.25 ± 0.13
BetaCrosslaps (ng/l)	709.65 ± 13.57	795.23 ± 24.21	876.86 ± 28.06
Serum calcium (mmol/l)	2.40 ± 0.01	2.39 ± 0.02	2.46 ± 0.03
Serum phosphate (mmol/l)	1.20 ± 0.01	1.19 ± 0.02	1.23 ± 0.03

Data are presented as Mean ± SE (SE – standard error of the mean)

*BMD* bone mineral density, *HT* hormone therapy (17ß estradiol/progesterone)

The studied women came from a Slovak southern region and, from a historical point of view, they could be considered as descendants of a mixed Hungarian-Slavic population. This territory has been an important Hungarian-Slavic contact zone for more than thousand years [24] and it has homogeneously merged the overlapping populations with a different cultural, linguistic and geographic origin.

### Clinical data acquisition

Personal and family history, age and life style habits were examined using a questionnaire (Additional file 1) that was completed by the subjects and reviewed by the qualified physician. BMI was calculated as weight in kilograms divided by height in meters squared. A prevalence (presence or absence) of total, femoral, radial, and spinal fragility fractures (also included compression fractures) in a period of last 5 years was diagnosed by clinical evaluation and using X-rays radiographs. A detailed personal history was considered to avoid counting traumatic fractures. BMD expressed by T-score and g/cm^2^ of femoral (BMD-FN) and lumbar spine vertebrae (BMD-LS) was measured at the femoral neck and at the lumbar spine (L2-L4) by dual energy X-ray absorptiometry (HOLOGIC Discovery DXA system). All women were tested with the same densitometer. Biochemical markers of bone remodeling included osteoformation and osteoresorption markers - bone isoenzyme of alkaline phosphatase (ALP; μkat/l), serum osteocalcin (OC; μg/l), serum beta CrossLaps (CTx; ng/l). The ALP was determined by immunoenzymatic assay (Beckman Coulter Access Ostase assay, Beckman Coulter), the OC and CTx were measured by electrochemiluminescence immunoassay with cobas e411 (Roche Diagnostics) within a diagnostic screening. Concentrations of serum calcium (mmol/l) and phosphate (mmol/l) were analysed by photometric assay with cobas c311 (Roche Diagnostics). All measurements were performed by accredited clinical laboratories in Nitra (Slovakia).

### Genetic analysis of the *ESR1* gene

**Fig. 1:**
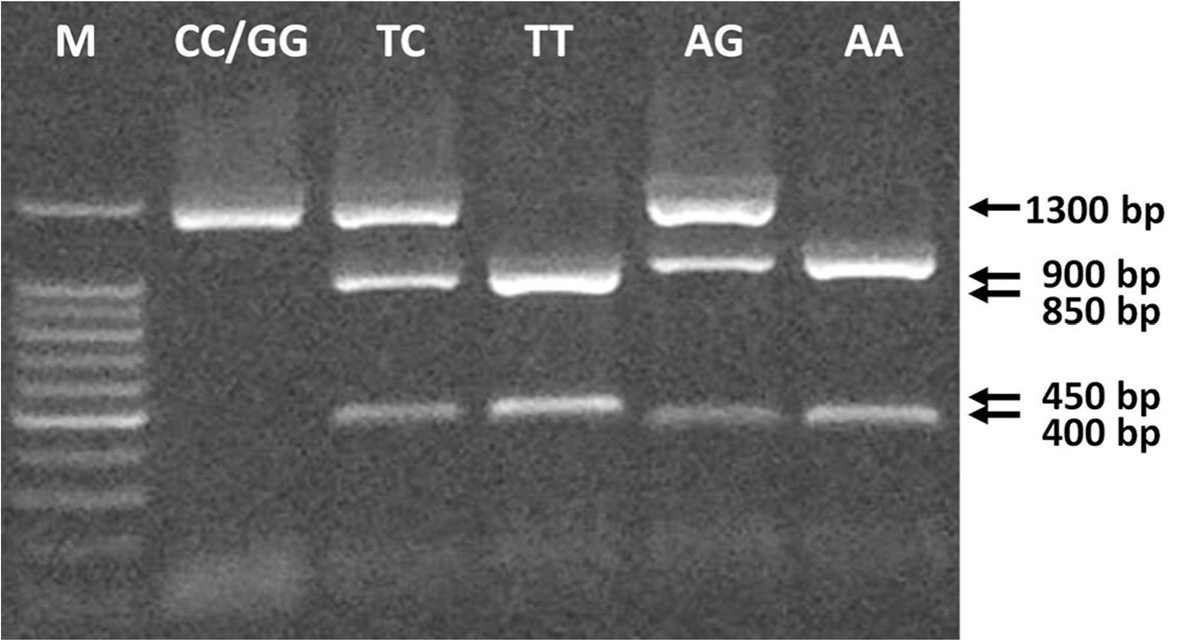
Representative results of *ESR1* genotypes detection. Lane M – 100 bp ladder; lane CC/GG – amplified *ESR1* gene (1300 bp) and CC or GG genotypes; the other lanes represent combinations of DNA fragments related to the rest *ESR1* genotypes

Genomic DNA was extracted from EDTA blood samples using the blood isolation kit (SiMax™ Genomic DNA Extraction Kit, China). DNA was amplified by PCR using primers according to Kobayashi et al. [11]. PCR was performed with the following steps: 95 °C for 5 min and then 94 °C for 30 s, 60 °C for 30 s, and 72 °C for 1 min. The PCR consisted of 35 cycles and it was completed by a final extension cycle at 72 °C for 7 min. The PCR product was a 1.3-kb long fragment including a part of intron 1 and exon 2 of the *ESR1* gene. After amplification, the PCR product was digested with XbaI and PvuII restriction endonucleases (Invitrogen) separately at 37 °C overnight and separated by electrophoresis in 2.0% agarose gel containing ethidium bromide. The gels were documented by DNR Bio-Imaging Systems (MiniBIS Pro, Israel). The “G” and “C” alleles indicate the absence of XbaI and PvuII restrictrion site, respectively, the “A” and “T” alleles indicate a presence of these restriction sites (Fig. 1).

### Pharmacogenetic study

Data from osteoporotic women, who received regular anti-osteoporotic therapy during 48 months, were analysed (Table 1). BMDs (BMD-FN and BMD-LS) were measured before and after the treatment period. The therapy types included application of hormone therapy (HT) of 17ß estradiol in combination with progesterone (1 mg/day for both; *N* = 76) or SERMs/raloxifene (60 mg/day; *N* = 64). During a treatment, all women received a supplementation of calcium (1000 mg/day) and vitamin D (800 IU/day).

### Statistical analysis

The data were summarized as Mean ± SE (Standard Error of the Mean) for quantitative variables and as frequencies for qualitative variables. Genotype distribution was tested for Hardy–Weinberg equilibrium using the chi-square test. The differences of quantitative variables among the genotypes were analysed in quantitative design by covariance analysis (General Linear Model procedure, GLM) after correction of the measurements for age and BMI. A dominant genetic model (TT vs. TC + CC for rs2234693; AA vs. AG + GG for rs9340799) was chosen according to calculations by SNPStats (Institut Català d’Oncologia). Possible interactions (significance interval) were tested using Johnson-Neyman procedure [25]. For evaluation of fracture prevalence Binary Logistic Regression with the genotype, age and BMI as covariates was used. The effect of genotypes on BMD change during a treatment was assessed by variance analysis for repeated measurements using GLM, where the evaluated BMD before and after treatment represented a repeat dependent variable and the individual genotypes were fixed effects. The BMD improvement within a genotype was tested by the same procedure but without between subject factors. Corrections for multiple testing of genotype effects were performed by Bonferroni correction. Statistical analysis was realized using SPSS software version 17.0 (SPSS Inc.; Chicago, IL, USA). The same software package was used to calculate the observed power of the association and pharmacogenetic studies. According to the relatively small sample size, the ideal power analysis parameters for our study were expected at 80% for the observed power with small to medium effect size. The *p*-value less than 0.05 was considered to be statistically significant.

## Results

In our studied group we found the highest frequencies of heterozygous genotypes for both polymorphisms (Table 2). The distribution of genotypes agreed with that expected according to the Hardy-Weinberg equilibrium. In addition, rs2234693 and rs9340799 polymorphisms in the *ESR1* gene were in linkage disequilibrium (χ^2^ = 363.56; *P* < 0.001). The frequencies of the haplotypes counted 0.52, 0.37, 0.10, and 0.01 for TA, CG, CA, and TG haplotypes, respectively.

**Table 2:** Distribution of *ESR1* genotypes and alelles

Polymorphism	Genotype	Number	Genotype frequency (%)	HWE *P* value	Alelle frequency
rs9340799	GG	52	15.2	χ2 = 0.209 *P* = 0.90	G = 0.38 A = 0.62
	AG	157	45.8		
	AA	134	39.0		
rs2234693	CC	73	21.3	χ2 = 0.188 *P* = 0.91	C = 0.47 *T* = 0.53
	CT	175	51.0		
	TT	95	27.7		

*HWE* Hardy-Weinberg equilibrium (the chi-square test value)

Associations of rs9340799 and rs2234693 genotypes with the osteoporosis-related characteristics are presented in Table 3. The results of statistical analysis for rs9340799 polymorphism showed that femoral and spinal BMD were significantly higher in women with the AA genotype in comparison with AG + GG genotypes (*P* < 0.05). No statistically significant difference between the rs9340799 genotypes was observed for other analysed traits (ALP, OC, CTx, Ca, P). Moreover, no association of rs2234693 genotype with BMD, biochemical markers of bone turnover and other serum parameters was found. A haplotype analysis revealed non-significant effects of TA and CG haplotypes on any of the analysed trait.

**Table 3:** Associations of the rs9340799 and rs2234693 genotypes with osteoporosis-related traits

Parameter	rs9340799:A > G genotypes		Sig. (*P* value)	Sig. Cov.	BMD difference
	AA *N* = 134	AG + GG *N* = 209			
BMD-FN (T-score)	−1.716 ± 0.044	−1.837 ± 0.035	0.035	A	0.120 ± 0.570
BMD-FN (g/cm^2^)	0.587 ± 0.07	0.566 ± 0.06	0.035	A	0.020 ± 0.010
BMD-LS (T-score)	−2.262 ± 0.062	−2.432 ± 0.049	0.033	A, B	0.170 ± 0.079
BMD-LS (g/cm^2^)	0.741 ± 0.07	0.723 ± 0.05	0.033	A, B	0.018 ± 0.008
ALP	0.493 ± 0.057	0.588 ± 0.046	NS	A, B	
OC	3.833 ± 0.086	3.853 ± 0.069	NS	A	
CTx	691.442 ± 21.246	721.324 ± 16.992	NS	A	
sCa	2.400 ± 0.017	2.399 ± 0.014	NS	A	
sP	1.191 ± 0.015	1.206 ± 0.012	NS	B	
Parameter	rs2234693:T > C genotypes		Sig. (P value)	Sig. Cov.	BMD difference
	TT *N* = 95	TC + CC *N* = 248			
BMD-FN (T-score)	−1.714 ± 0.053	−1.818 ± 0.032	NS	A	
BMD-FN (g/cm^2^)	0.585 ± 0.09	0.570 ± 0.05	NS	A	
BMD-LS (T-score)	−2.277 ± 0.073	− 2.400 ± 0.045	NS	A, B	
BMD-LS (g/cm^2^)	0.739 ± 0.08	0.726 ± 0.05	NS	A, B	
ALP	0.582 ± 0.068	0.547 ± 0.042	NS	A, B	
OC	3.926 ± 0.102	3.814 ± 0.063	NS	A	
CTx	696.002 ± 25.214	714.878 ± 15.592	NS	A	
sCa	2.394 ± 0.020	2.401 ± 0.013	NS	A	
sP	1.170 ± 0.018	1.208 ± 0.011	NS		

Data are presented as Estimated Marginal Mean ± SE (SE – standard error of the mean); values are adjusted for age and *BMI* BMD-FN – femoral neck BMD (T-score and g/cm^2^), *BMD-LS* – lumbal spine BMD (T-score and g/cm^2^), *ALP* – bone isoenzyme of alkaline phosphatase (μkat/l), *OC* - osteocalcin (μg/l), *CTx* - BetaCrosslaps (ng/l), *sCa* - serum calcium (mmol/l), *sP* - serum phosphate (mmol/l), *Sig.* – significance of GLM/BMD differences, *NS* – non-significant GLM, *P* values determine significant GLM/BMD differences (*P* < 0.05), *Sig. Cov*. - significance of covariates, *A* – significant (*P* < 0.05) covariate Age, *B* - significant (*P* < 0.05) covariate BMI

None of the polymorphisms of the *ESR1* genotypes had an effect on fracture prevalence (Table 4). Femoral fractures were not included in the analysis because of a small number of femoral fracture carriers (*N* = 4).

**Table 4:** The effects of the rs9340799 and rs2234693 genotypes on fracture prevalence

Fracture location	Genotypes	Presence of fractures	Absence of fractures	*P* value	OR	95% CI
rs9340799:A > G genotypes						
Spinal	GG	12	40	0.744	0.869	0.373–2.024
	AG	43	114	0.464	0.764	0.437–1.458
	AA	36	98			
Radial	GG	8	44	0.501	0.722	0.279–1.868
	AG	27	130	0.371	0.735	0.375–1.442
	AA	21	113			
Total	GG	13	39	0.913	1.046	0.469–2.330
	AG	48	109	0.632	0.871	0.495–1.533
	AA	42	92			
rs2234693:T > C genotypes						
Spinal	CC	17	56	0.861	0.930	0.414–2.092
	TC	48	127	0.759	0.903	0.472–1.729
	TT	26	69			
Radial	CC	10	63	0.856	0.918	0.363–2.322
	TC	31	144	0.501	0.779	0.377–1.610
	TT	15	80			
Total	CC	18	55	0.858	1.073	0.497–2.315
	TC	56	119	0.583	0.843	0.458–1.552
	TT	29	66			

The total number values count a presence of any fracture in an individual, *OR* - the odds ratio, *CI* - confidence interval, AA and TT genotypes were set as baseline categories in a regression model; femoral fractures were not evaluated

**Fig. 2:**
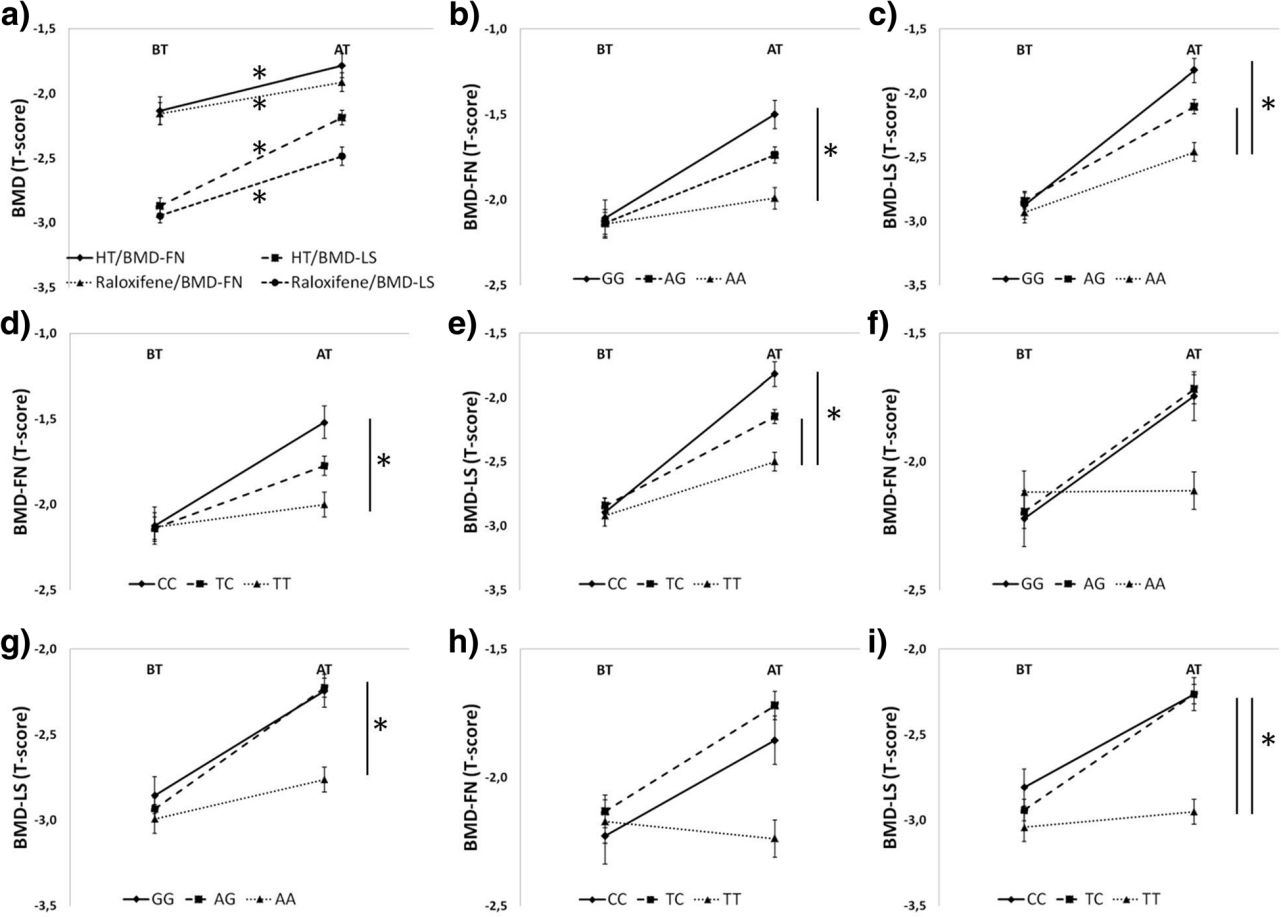
Effects of the treatment on BMD change. **a** effect of a treatment type on BMD-FN and BMD-LS change; **b-e** effect of HT on BMD-FN and BMD-LS change according to the rs2234693 and rs9340799 genotypes; **f-i** effect of raloxifene treatment on BMD-FN and BMD-LS change according to the rs2234693 and rs9340799 genotypes; BT – before treatment; AT – after treatment; * indicates significant differences (*P* < 0.05)

The findings from the pharmacogenetic analysis showed that both evaluated treatment types had a significant effect on positive BMD change after 48 months of treatment (Table 5; Fig. 2). Within HT, an increase in T-score of 0.347 ± 0.043 and 0.687 ± 0.057 was found for BMD-FN and BMD-LS, respectively. Raloxifene increased the T-score by 0.242 ± 0.070 and 0.463 ± 0.063 in BMD-FN and BMD-LS, respectively. The treatment efficiency of the therapies ranged from + 5.2 to + 11.3% of BMD increase. However, when considering the effects of the *ESR1* gene, significant differences in treatment efficiency also between *ESR1* genotypes were revealed (Tables 6, 7; Fig. 2). Significant changes were found in femoral neck, as well as in lumbar spine BMD. Among HT treated women, the subjects with GG genotype of rs9340799 had significantly better response to HT than those with AA genotype in both, BMD-FN (P<0.05) and BMD-LS (*P*<0.01). In these cases, the T-scores were different by 0.262 ± 0.103 and 0.345 ± 0.100 at BMD-FN and BMD-LS, respectively. Moreover in BMD-LS, the women with AA genotype responded poorly to the therapy when compared also with AG genotype (− 0.221 ± 0.077 of T-score; *P*<0.05). Within the rs2234693 genotypes, individuals with CC (+ 0.354 ± 0.094 of T-score; *P* ≤ 0.001) and TC (+ 0.215 ± 0.080 of T-score; *P*<0.05) genotypes had better response to HT in BMD-LS in comparison with TT genotype carriers. Despite these differences, all genotypes (except for TT in BMD-FN) showed significant increase in BMD during HT treatment, counting from + 3.4 to + 17.4%.

**Table 5:** The effect of a treatment type on BMD change

Treatment type	Skeletal site	BMD before treatment	BMD after treatment	BMD difference after treatment	Sig. (*P* value)
HT	FN T-score	−2.132 ± 0.044	−1.784 ± 0.041	0.347 ± 0.043	0.001
	FN BMD	0.603 ± 0.06	0.649 ± 0.05	0.046 ± 0.05 (+ 7.3%)	0.001
	LS T-score	−2.871 ± 0.044	− 2.184 ± 0.048	0.687 ± 0.057	0.001
	LS BMD	0.674 ± 0.005	0.755 ± 0.005	0.081 ± 0.006 (+ 11.3%)	0.001
raloxifene	FN T-score	−2.155 ± 0.059	−1.913 ± 0.078	0.242 ± 0.070	0.002
	FN BMD	0.597 ± 0.008	0.635 ± 0.010	0.038 ± 0.009 (+ 5.2%)	0.002
	LS T-score	−2.947 ± 0.054	−2.484 ± 0.070	0.463 ± 0.063	0.001
	LS BMD	0.670 ± 0.007	0.725 ± 0.008	0.055 ± 0.007 (+ 7.7%)	0.001

BMD of femoral neck (FN) and lumbal spine (LS) is expressed as Estimated Marginal Mean ± SE (SE – standard error of the mean) of T-score (FN and LS T-score) and g/cm^2^ (FN and LS BMD); HT – hormone therapy (17ß estradiol/progesterone); Sig. – significance of BMD difference after treatment, P values determine significant differences (*P* < 0.05)

**Table 6:** BMD changes after hormone therapy in relation to the rs9340799 and rs2234693 genotypes

Genotypes	Skeletal site	BMD before treatment	BMD after treatment	BMD difference after treatment	GLM Sig. (*P* value)	PC Sig. (*P* value)
rs9340799:A > G genotypes						
GG	FN T-score	−2.108 ± 0.106	−1.500 ± 0.082	0.608 ± 0.090	0.001	
	FN BMD	0.605 ± 0.013	0.678 ± 0.010	0.073 ± 0.011 (+ 12.6%)	0.001	
AG	FN T-score	−2.138 ± 0.063	−1.738 ± 0.049	0.400 ± 0.061	0.001	
	FN BMD	0.601 ± 0.008	0.649 ± 0.006	0.048 ± 0.007 (+ 8.4%)	0.001	
AA	FN T-score	−2.141 ± 0.082	−1.991 ± 0.063	0.150 ± 0.066	0.035	
	FN BMD	0.601 ± 0.010	0.619 ± 0.008	0.018 ± 0.008 (+ 3.4%)	0.035	
GG-AG	FN T-score			0.134 ± 0.952	0.042 for FN T-score and FN BMD	NS
	FN BMD			0.016 ± 0.011		NS
GG-AA	FN T-score			0.262 ± 0.103		0.035
	FN BMD			0.031 ± 0.012		0.035
AG-AA	FN T-score			0.128 ± 0.795		NS
	FN BMD			0.015 ± 0.010		NS
GG	LS T-score	−2.877 ± 0.109	−1.823 ± 0.094	1.054 ± 0.053	0.001	
	LS BMD	0.675 ± 0.012	0.788 ± 0.010	0.113 ± 0.006 (+ 16.9%)	0.001	
AG	LS T-score	−2.843 ± 0.064	− 2.105 ± 0.056	0.738 ± 0.073	0.001	
	LS BMD	0.679 ± 0.007	0.758 ± 0.006	0.079 ± 0.008 (+ 11.9%)	0.001	
AA	LS T-score	−2.932 ± 0.083	− 2.459 ± 0.073	0.473 ± 0.125	0.001	
	LS BMD	0.669 ± 0.009	0.720 ± 0.008	0.051 ± 0.013 (+ 8.3%)	0.001	
GG-AG	LS T-score			0.124 ± 0.092	0.002 for FN T-score and FN BMD	NS
	LS BMD			0.013 ± 0.010		NS
GG-AA	LS T-score			0.345 ± 0.100		0.003
	LS BMD			0.037 ± 0.011		0.003
AG-AA	LS T-score			0.221 ± 0.077		0.014
	LS BMD			0.024 ± 0.008		0.014
rs2234693:T > C genotypes						
CC	FN T-score	−2.124 ± 0.093	−1.518 ± 0.072	0.606 ± 0.081	0.001	
	FN BMD	0.603 ± 0.011	0.676 ± 0.09	0.073 ± 0.010 (+ 12.6%)	0.001	
TC	FN T-score	−2.139 ± 0.064	−1.772 ± 0.049	0.367 ± 0.058	0.001	
	FN BMD	0.601 ± 0.008	0.645 ± 0.006	0.044 ± 0.007 (+ 7.7%)	0.001	
TT	FN T-score	−2.132 ± 0.088	−2.000 ± 0.068	0.132 ± 0.077	NS	
	FN BMD	0.602 ± 0.011	0.618 ± 0.008	0.016 ± 0.009 (+ 3.0%)	NS	
CC-TC	FN T-score				NS for FN T-score and FN BMD	
	FN BMD					
CC-TT	FN T-score					
	FN BMD					
TC-TT	FN T-score					
	FN BMD					
CC	LS T-score	−2.894 ± 0.095	−1.818 ± 0.079	1.077 ± 0.067	0.001	
	LS BMD	0.673 ± 0.010	0.789 ± 0.008	0.115 ± 0.007 (+ 17.4%)	0.001	
TC	LS T-score	−2.844 ± 0.065	−2.147 ± 0.054	0.697 ± 0.064	0.001	
	LS BMD	0.679 ± 0.007	0.753 ± 0.006	0.075 ± 0.007 (+ 11.2%)	0.001	
TT	LS T-score	−2.921 ± 0.090	− 2.500 ± 0.075	0.421 ± 0.146	0.010	
	LS BMD	0.670 ± 0.010	0.715 ± 0.008	0.045 ± 0.016 (+ 7.6%)	0.010	
CC-TC	LS T-score			0.140 ± 0.083	0.001 for FN T-score and FN BMD	NS
	LS BMD			0.015 ± 0.009		NS
CC-TT	LS T-score			0.354 ± 0.094		0.001
	LS BMD			0.038 ± 0.010		0.001
TC-TT	LS T-score			0.215 ± 0.080		0.026
	LS BMD			0.023 ± 0.009		0.026

BMD of femoral neck (FN) and lumbal spine (LS) is expressed as Estimated Marginal Mean ± *SE* (SE – standard error of the mean) of T-score (FN and LS T-score) and g/cm^2^ (FN and LS BMD), *GLM Sig*. – significance of GLM, *PC Sig*. – significance of pairwise comparisons, *NS* – non-significant GLM/BMD differences, *P* values determine significant BMD differences (*P* < 0.05)

**Table 7:** BMD changes after raloxifene therapy in relation to the rs9340799 and rs2234693 genotypes

Genotype	Skeletal site	BMD before treatment	BMD after treatment	BMD difference after treatment	GLM Sig. (*P* value)	PC Sig. (*P* value)
GG	FN T-score	−2.222 ± 0.160	−1.744 ± 0.201	0.478 ± 0.105	0.002	
	FN BMD	0.591 ± 0.019	0.649 ± 0.024	0.057 ± 0.013 (+ 10.2%)	0.002	
AG	FN T-score	−2.196 ± 0.100	−1.717 ± 0.126	0.478 ± 0.157	0.006	
	FN BMD	0.595 ± 0.012	0.652 ± 0.015	0.057 ± 0.019 (+ 9.9%)	0.006	
AA	FN T-score	−2.119 ± 0.086	−2.113 ± 0.108	0.006 ± 0.058	NS	
	FN BMD	0.604 ± 0.010	0.604 ± 0.013	0.001 ± 0.007 (+ 0.46%)	NS	
GG-AG	FN T-score				NS for FN T-score and FN BMD	
	FN BMD					
GG-AA	FN T-score					
	FN BMD					
AG-AA	FN T-score					
	FN BMD					
GG	LS T-score	−2.856 ± 0.145	−2.244 ± 0.167	0.611 ± 0.082	0.001	
	LS BMD	0.677 ± 0.016	0.743 ± 0.018	0.065 ± 0.009 (+ 9.7%)	0.001	
AG	LS T-score	−2.935 ± 0.091	−2.226 ± 0.105	0.709 ± 0.087	0.001	
	LS BMD	0.669 ± 0.010	0.745 ± 0.011	0.076 ± 0.009 (+ 11.5%)	0.001	
AA	LS T-score	−2.994 ± 0.078	−2.765 ± 0.090	0.229 ± 0.094	0.021	
	LS BMD	0.663 ± 0.008	0.687 ± 0.010	0.025 ± 0.010 (+ 4.1%)	0.021	
GG-AG	LS T-score			0.030 ± 0.161	0.016 for FN T-score and FN BMD	NS
	LS BMD			0.003 ± 0.017		NS
GG-AA	LS T-score			0.329 ± 0.155		NS
	LS BMD			0.035 ± 0.017		NS
AG-AA	LS T-score			0.299 ± 0.113		0.028
	LS BMD			0.032 ± 0.012		0.028
CC	FN T-score	−2.227 ± 0.145	−1.855 ± 0.177	0.373 ± 0.093	0.002	
	FN BMD	0.591 ± 0.017	0.635 ± 0.021	0.045 ± 0.092 (+ 7.8%)	0.002	
TC	FN T-score	−2.132 ± 0.086	−1.719 ± 0.106	0.413 ± 0.124	0.002	
	FN BMD	0.602 ± 0.010	0.652 ± 0.013	0.050 ± 0.015 (+ 8.6%)	0.002	
TT	FN T-score	−2.171 ± 0.105	−2.238 ± 0.128	0.067 ± 0.060	NS	
	FN BMD	0.597 ± 0.013	0.589 ± 0.015	−0.008 ± 0.007 (−1.0%)	NS	
CC-TC	FN T-score				NS for FN T-score and FN BMD	
	FN BMD					
CC-TT	FN T-score					
	FN BMD					
TC-TT	FN T-score					
	FN BMD					
CC	LS T-score	−2.809 ± 0.130	−2.264 ± 0.140	0.546 ± 0.092	0.001	0.001
	LS BMD	0.682 ± 0.014	0.741 ± 0.015	0.058 ± 0.010 (+ 8.6%)	0.001	0.001
TC	LS T-score	−2.942 ± 0.077	−2.265 ± 0.084	0.677 ± 0.074	0.001	0.001
	LS BMD	0.668 ± 0.008	0.741 ± 0.009	0.073 ± 0.008 (+ 11.0%)	0.001	0.001
TT	LS T-score	−3.043 ± 0.094	−2.952 ± 0.102	0.091 ± 0.114	NS	NS
	LS BMD	0.657 ± 0.010	0.667 ± 0.011	0.010 ± 0.012 (+ 2.0%)	NS	NS
CC-TC	LS T-score			0.067 ± 0.137	0.001 for FN T-score and FN BMD	NS
	LS BMD			0.007 ± 0.015		NS
CC-TT	LS T-score			0.461 ± 0.145		0.007
	LS BMD			0.049 ± 0.016		0.007
TC-TT	LS T-score			0.394 ± 0.110		0.002
	LS BMD			0.042 ± 0.012		0.002

BMD of femoral neck (FN) and lumbal spine (LS) is expressed as Estimated Marginal Mean ± *SE* (SE – standard error of the mean) of T-score (FN and LS T-score) and g/cm^2^ (FN and LS BMD); GLM Sig. – significance of GLM, *PC Sig.* – significance of pairwise comparisons, *NS* – non-significant GLM/BMD differences, *P* values determine significant BMD differences (*P* < 0.05)

The effect of the *ESR1* gene on raloxifene therapy was reported only in relation to BMD-LS. Subjects with AA genotype had significantly worse response to raloxifene, counting − 0.299 ± 0.113 of T-score (P<0.05), when compared with AG genotype. Finally, patients with TT genotype showed 0.394 ± 0.110 and 0.461 ± 0.145 lower T-score in BMD-LS (P<0.01) than those with the TC and CC genotypes, respectively. No changes were detected in the femoral neck BMD in relation to the raloxifene therapy.

## Discussion

At older ages, osteoporosis may be the cause of diminished life quality, decreased functional independence, increased morbidity and, even sometimes, mortality. Genetic research helps to reveal responsible genetic factors, which can expand our possibilities in the treatment of the disease or an identification of individuals at risk.

Our results point to similar genetic variability in rs2234693 and rs9340799 polymorphisms as in other Caucasian populations [9, 19, 26, 27]. Differences in genotype distribution of both polymorphisms can be found between Caucasian and other populations. Data from Asian populations [11, 28–30] showed differential range of allele and genotype frequency. The rs2234693 genotype distribution moves in the range of 14.0–19.3%, 43.6–54.8%, and 29.4–39.1% for CC, TC, and TT genotypes, respectively. The rs9340799 genotype distribution counts 3.5–7.0%, 27.4–35.0%, and 58.6–67.2% for GG, AG, and AA genotype, respectively.

In our study, an association between rs9340799 polymorphism of the *ESR1* gene and BMD was found. The AA genotype individuals had a significantly higher BMD values compared to AG + GG genotypes. Previous association studies, involving different populations, have produced inconsistent results. In most studies of Caucasian populations, significant associations of rs2234693 and rs9340799 polymorphisms and BMD have not been recorded [31, 32]. Higher BMD-FN was revealed in women from the United States, who were homozygous for C and G alleles of the *ESR1* gene [13]. Van Meurs et al. [10] investigated the impact of rs2234693/rs9340799 haplotypes on BMD in a large population sample of white postmenopausal women, and found a significant association of the TA haplotype with a decreased BMD-LS, whereas the CG haplotype was associated with an increased BMD-LS; no association was found with BMD-FN. In addition, Albagha et al. [26] analysed white women from the United Kingdom, and found that only the CA haplotype was associated with lower values of BMD. Inconsistent results of association studies can be observable also in Asian populations [28–30]. A meta-analysis of 30 studies published by Ioannidis et al. [33] showed a positive effect of GG genotype on BMD and fracture risk, whereas rs2234693 polymorphism was not associated with these traits. In a recent review and meta-analysis of Zhu et al. [34] the authors found significant associations of *ESR1* polymorphisms with BMD in Caucasian women. The GG and AG genotypes were associated with increased FN BMD and FN Z value, respectively. These genotypes also had a higher LS Z value in comparison with AA genotype. CC genotype was associated with a low LS Z value, TC genotype in osteoporotic women was significantly correlated with low FN Z value. The discrepancies between different studies and populations can be explained by ethnic differences or higher variability in studied samples (peri-, pre-, post-menopausal women, multiple pregnancies, different sample size). Interactions between *ESR1* gene and other genetic polymorphisms should also be considered [29, 35].

We did not find any association between *ESR1* genotypes and a presence of fractures. Within a meta-analysis of Tang et al. [36], rs2234693/T allele was strongly identified as a significant risk factor for hip fracture among Caucasian populations, but not in Asian ones. However, in addition to BMD, genetic factors may contribute to fracture risk through mechanisms other than bone mass. These factors can include various skeletal characteristics like bone size and shape, cortical porosity, trabecular microarchitecture, and osteocyte cell function that may not be well captured by BMD measurements alone [37]. Some studies point to the importance of bone microdamage accumulation in the initiation of bone resorption and remodeling [38]. It would be perspective to include these factors into analyses in relation to fractures. Moreover, BMD changes have a long-term character, while bone turnover markers can directly infer about processes in bone tissue (formation/resorption). In our study, no differences in bone turnover markers between *ESR1* genotypes were observed. In recent years, large genome-wide association studies (GWAS) have brought new insights into the genetics of osteoporosis. Some of the studies replicated previously reported candidate genes (including the *ESR1* gene) in association with BMD and fracture risk [3]. The largest meta-analysis [39] included 17 genome-wide association studies with individuals of European and East Asian ancestry and identified 56 loci (with 24 reported previously) associated with BMD variation and 14 loci associated with risk of fracture. Further studies should be directed towards polymorphisms that have shown significant results in genome-wide association studies to evaluate their effect in specific populations.

Pharmacogenetic research has a potential to allow efficacious treatments, with consequent better chances for the patient health and reduced economic loss [40]. In our study, the effect of rs2234693 and rs9340799 polymorphisms on antiosteoporotic treatment efficiency was revealed. Similar outcomes, where genotypes with C or G alleles were associated with greater sensitivity to HT, have been documented in other studies. Salmen et al. [19] analysed Finnish postmenopausal women during 5-years of HT. They found that women with the TT genotype had a greater fracture risk in comparison with C allele carriers. In study of Giguere et al. [41], women with combined *VDR*-bb/*ESR1*-CC genotype who received HT for more than 5 years, had a 21% greater ultrasound heel stiffness index z score (comparable with BMD scores) than those with the same genotype receiving HT for less than 5 years. The study included postmenopausal women of French-Canadian origin. Rapuri et al. [20] reported significantly higher BMD response to HT treatment in women with the CC genotype compared to TT genotype. Similar findings with a positive effect of the C allele on vertebral BMD were found in the study by Ongphiphadhanakul et al. [21]. Subjects consisted of Thai post-menopausal women and the effect was not found on femoral BMD. Greater increase in lumbar spine BMD was recorded in CC genotypes in postmenopausal Japanese women [12]. No differences in HT efficiency have also been demonstrated in other studies [42, 43].

SERMs have the ability to bind to the estrogen receptor and act as a receptor agonist or antagonist in a tissue-specific manner. Raloxifene (the estrogen receptor agonist in bone) was the first SERM approved for the prevention and treatment of postmenopausal osteoporosis [44]. According to our results, individuals with CC or TC genotypes of rs2234693 and AG genotype of rs9340799 better responded on raloxifene therapy with higher BMD-LS changes in comparison with homozygous TT or AA genotypes. Similarly, postmenopausal osteoporotic women with the CC or AA genotypes on chronic hemodialysis exhibited a better lumbar spine BMD response in a study by Heilberg et al. [45]. Higher increase in total hip BMD was also noticed in postmenopausal women with osteoporosis carrying CC or TC genotypes [46]. Positive efficacy of raloxifene on BMD was also monitored in relation to other genes [47, 48].

Focusing on the percent change in BMD, a very high treatment efficacy in our study is remarkable, reaching up to 11.3 and 7.7% for HT and raloxifene in LS site, respectively. A meta-analysis of Wells et al. [49] showed a BMD-LS gain of 8% using high-dose estrogen (equivalent to 0.9 mg Premarin) during 2 years. The BMD-LS improvement after raloxifene therapy usually reaches around 2.5% after 2 years [50]. Several factors may contribute to the differences between studies. From the point of view of our study, we can consider especially limited sample size, differences from other studies in BMD baseline, population composition, or inclusion criteria (e.g. adequacy of calcium/vitamin D intake, previous anti-resorptive treatment). The effect of a therapy was also found to be a dose and time dependent.

Considering limitations of our study, the small sample size seems to be the most important. Despite the ability to calculate optimal sample size, the number of observations is often dependent on the existing economic and human resources or the time available for carrying out the study. The observed power for our study, where the model was significant, ranged from 69 to 73% and from 78 to 94% for association and pharmacogenetic analyses, respectively. Moreover, the revealed effects of the polymorphisms cannot be confirmed on the molecular level. The mechanisms by which the polymorphisms may influence bone mass are still not clear, since these polymorphisms lie in an intronic area of the gene. However, a study of Herrington et al. [51] showed that a functional binding site for the transcription factor B-myb is absent with the T allele, which, in turn, may reduce *ESR1* transcription rates or produce a functionally different ESR1 isoform. It has also been demonstrated that the *ESR1* gene expression can be regulated by epigenetic mechanisms [52]. Moreover, there is still the possibility that both polymorphisms are only linkage markers and the effect itself is caused by another, closely related region of the *ESR1* gene. In any case, all the mechanisms may also be the cause of different impacts of individual polymorphisms on the analysed parameters. Other limitations can involve gene-gene and gene-environment interactions, or epigenetic factors which could influence the pharmacodynamics and pharmacokinetics of individual drug response [18]. Nevertheless, the pharmacogenetic research is promising, especially for osteoporosis, that require long-term treatments and where different therapy types exist to be alternatively chosen.

## Conclusion

We found that rs9340799 polymorphism may contribute to decreased BMD in postmenopausal women from southern Slovakia, whereas rs2234693 polymorphism did not affect any of the analyzed parameters. The *ESR1* gene was not significantly related to fracture prevalence. Our study also demonstrated the effect of both *ESR1* gene polymorphisms on the effectiveness of HT (17ß estradiol/progesterone), as well as SERMs/raloxifene therapies with poorer response in patients with rs2234693/TT and rs9340799/AA genotypes. The results can contribute to a more comprehensive insight to the genetics and pharmacogenetics of osteoporosis. The evaluation of effects of previously revealed candidate genes in specific populations may get closer to the practical use of results in predictive genetics and personalized medicine.

## Additional file

Additional file 1: Anamnestic questionnaire for osteological examination. Questionnaire form for personal and family history, age, and life style habits data acquisition. (PDF 332 kb)

## Abbreviations

ALP: Bone isoenzyme of alkaline phosphatase
BMD: Bone mineral density
BMD-FN: Femoral neck bone mineral density
BMD-LS: Lumbar spine bone mineral density
BMI: Body mass index
BPs: Bisphosphonates
CI: Confidence interval
CTx: Beta CrossLaps
*ESR1*: Estrogen receptor 1 gene
FN: Femoral neck
GLM: General linear model
GWAS: Genome-wide association studies
HT: Hormone therapy
HWE: Hardy-Weinberg equilibrium
LS: Lumbar spine
NS: Non-significant
OC: Osteocalcin
OR: The odds ratio
PC Sig.: Significance of pairwise comparisons
PCR: Polymerase chain reaction
sCa: serum calcium
SE: Standard error of the mean
SERMs: Selective estrogen receptor modulators
Sig. Cov.: Significance of covariates
Sig.: Significance
sP: serum phosphate
*VDR*: Vitamin D receptor gene
