# Supplementary material for: The estrogen receptor 1 gene affects bone mineral density and osteoporosis treatment efficiency in Slovak postmenopausal women
Source: BMC Med Genet. 2018 Sep 21;19:174. doi: 10.1186/s12881-018-0684-8 (PMC6150952; doi:10.1186/s12881-018-0684-8)
Supplement: Supplementary file 1 — Anamnestic questionnaire for osteological examination. Questionnaire form for personal and family history, age, and life style habits data acquisition. (PDF 332 kb) [file 12881_2018_684_MOESM1_ESM.pdf]

ANAMNESTIC QUESTIONNAIRE FOR OSTEOLOGICAL EXAMINATION

(fill in the form with text, numbers or check ☒ that apply)

Name: ________________________________ Date: __________________

Primary care physician____________________________

A. General Information

1. Your gender: ☐ Male ☐ Female
2. Date of your birth: _______________
3. Your current height: _________cm and weight:_______ kg
   What was your tallest height (at around the age of 30)? _________ cm
4. Your marital status: ☐ Single ☐ Married ☐ Divorced or Widowed
5. Education level : ☐ Elementary/primary ☐ High school/secondary school
   ☐ College or university

B Bone health history

1. Does osteoporosis run in your family? ☐ Yes ☐ No
   If YES, who suffered? ☐ Mother ☐ Father ☐Other(s)______________________
2. Has either of your parents broken a bone after the age of 50? ☐ Yes ☐ No
   If YES, who suffered?
   ☐ Mother What bone(s) did she break? ____________________ At what age? ______
   ☐ Father What bone(s) did he break? ______________________ At what age? ______
3. Have you ever had a densitometry (bone mineral density, BMD) test done? ☐ Yes ☐ No
   If YES, what was the T-score? __________ Date of bone density scan ___________
4. Have you ever broken a bone? ☐ Yes ☐ No
   If YES, what bone(s) did you break? ______________________ At what age? ______
   If YES, how did you break the bone(s)? ☐ Low impact injury (stumble, slip or simple fall)
   ☐ Fall down more than 1 stair or off a ladder ☐ Accident (car or motorcycle accident)
   ☐ High impact sports injury ☐ Other: ___________________________________
5. Have you been diagnosed and treated by a doctor of the following specialisation?
   ☐ rheumatology ☐ orthopedics ☐ gynecology ☐ oncology ☐ endocrinology
   ☐ diabetology ☐ cardiology ☐ general practitioner ☐ none of these
6. Have you ever had any serious illness? ☐ Yes ☐ No
   If YES, please specify: ____
7. Have you ever taken steroids, corticoids, or hormones? ☐ Yes ☐ No
   If YES, provide a list of medications and time of treatments: ____________________________
8. Do you take any of medications for osteoporosis treatment? ☐ Yes ☐ No
   If YES, provide a list of all medications that you take:
   ____

C Activity and lifestyle

1. Your occupation is: ☐ Sedentary ☐ With a movement during a work
   ☐ With a very active movement during a work
2. Would you describe yourself as a physically active individual? ☐ No ☐ Yes, all my life
   ☐ Yes, when I was younger ☐ Yes, more recently

3. How many kilometers do you walk on a daily basis? _________ km
4. Your average daily housework time: ________ hours
5. Your daily sit-in time: ________ hours (including watching computers, TV)
6. Do you do any regular exercises? ☐ No, never go to exercise ☐ 1-2 times / week
   ☐ 3-5 times / week ☐ > 5 times / week
   If YES, what type of exercise do you do? ____
7. Do you smoke cigarettes? ☐ Never ☐ Yes, average ___ cigarettes/day
   ☐ Used to, I stopped ____ years ago
8. Do you drink alcohol? ☐ No ☐ Yes, average ___ drinks/day
   ☐ Used to, I stopped ____ years ago
9. Do you drink coffee: ☐ No ☐ Yes, average __ drinks/day
   ☐ Used to, I stopped ____ years ago
10. Do you consume dairy products daily? ☐ No ☐ 1-2 servings daily ☐ 3-4 servings daily
   ☐ 5 or more servings daily
   If YES, what type of products do you consume most often? ______________________________

D For women
1. Age at your first menstrual cycle: ________ years.
2. Your menstrual cycles are/were: ☐ regular with menstrual cycle ___ days ☐ irregular
3. Have you gone through menopause? ☐ Yes ☐ No
   If YES, what was age or date of last menstrual period? _____________________
4. Number of pregnancies:_______ If none, was this due to infertility? ☐ Yes ☐ No
5. Number of births:___________ Your age at time of your first delivery:_________
6. Have you had a hysterectomy? ☐ Yes, at age of ______ ☐ No
7. Have you had yours ovaries removed? ☐ No ☐ Both, at age of ______
   ☐ One, at age of ______
8. Have you ever taken menopausal hormones? ☐ Yes, ___________ years long ☐ No
9. Have you used hormone replacement/estrogen therapy? ☐ Yes, from_______ to ________
   ☐ No
